# The quality of life of children with neurodevelopmental disorders and their parents during the Coronavirus disease 19 emergency in Japan

**DOI:** 10.1038/s41598-021-82743-x

**Published:** 2021-02-15

**Authors:** Riyo Ueda, Takashi Okada, Yosuke Kita, Yuri Ozawa, Hisami Inoue, Mutsuki Shioda, Yoshimi Kono, Chika Kono, Yukiko Nakamura, Kaoru Amemiya, Ai Ito, Nobuko Sugiura, Yuichiro Matsuoka, Chinami Kaiga, Masaya Kubota, Hiroshi Ozawa

**Affiliations:** 1 Department of Developmental Disorders, National Institute of Mental Health, National Center of Neurology and Psychiatry, 4-1-1 Ogawahigashi-Cho, Kodaira, Tokyo 187-8553 Japan; 2 Department of Child Neurology, Shimada Ryoiku Center Hachioji, Tokyo, Japan; 3 Mori Arinori Center for Higher Education and Global Mobility, Hitotsubashi University, Tokyo, Japan; 4 Cognitive Brain Research Unit, Faculty of Medicine, University of Helsinki, Helsinki, Finland

**Keywords:** Health care, Medical research

## Abstract

This study aimed to reveal how the COVID-19 stay-at-home period has affected the quality of life (QOL) of children with neurodevelopmental disorders and their parents and to identify possible factors that enabled them to maintain their QOL. We enrolled 136 school-aged children (intellectual quotient ≥ 50) and their parents and administered QOL questionnaires to assess the maladaptive behavior of the children; depression, anxiety, and stress of the parents; and activities of their daily lives. The relationship between their QOL and clinical features was examined. The decrease in QOL of children and parents was associated with the mother’s limited job flexibility. Decreased QOL was also associated with changes in the sleep rhythms of the children. Maladaptive behaviors in children were associated with parental stress. However, maintained QOL of some families who faced these same conditions of job stress and sleep disorders was associated with less parental stress, less parental depression and anxiety, and milder maladaptive behavior in children. Both mothers with limited job flexibility and changes in the sleep rhythm of children were associated with reduced QOL of children and their parents. Low parental stress was associated with decreased maladaptive behavior in children and with maintained QOL of the family.

## Introduction

People around the world have experienced profound changes in their lifestyles and relationships due to the coronavirus disease 2019 (COVID-19) pandemic and the requirements that individuals stay at home and practice social distancing. The disease spread rapidly throughout Japan, and an emergency declaration was issued by the prime minister on April 7, 2020. Citizens were mandated to stay at home and refrain from outside activities until May 25. School-aged children had been sent home even earlier, on March 2. Parents faced increased stressors if they had limited job flexibility, suffered job loss and reduced income, or had greatly increased responsibilities within the home.

Compared to typically developing children (TDC), children with neurodevelopmental disorders (NDDs) are more likely to suffer mental and physical difficulties during a disaster because of the unpredictable changes around them and alterations to their routines^1–3^. In recent studies, caregivers in Italy observed that children with autism spectrum disorder (ASD) engaged in more intense and frequent disruptive behavior during the COVID-19 quarantine^2^. Their parents were prone to depression and anxiety^4^ as they confronted difficulties in structuring their ASD children’s daily activities, especially free time^2^.

Quality of life (QOL) describes an individual’s subjective perception of their position in life, as evidenced by their physical, psychological, and social functioning^5^. The QOL of parents was greatly reduced by stress and the lack of coping skills in the face of increasingly aggressive behavior in their NDD children^5,6^.

As COVID-19 persists worldwide, it presents a different set of challenges compared to other natural disasters. The outside relationships an NDD child might have with schools and people in the community are weakened, and the child is exposed to more time with the family and social media.

Two primary lifestyle factors change the QOL of most children with NDD: The first is difficulties with their sleep cycles, which are associated with not only children’s mental problem but also poorer parent mental health and higher parenting stress^7^. The second is the parents’ work patterns and concerns about financial stability^8,9^. The stress that parents carry from their jobs impairs their childcare skills and affects the well-being of the children^9^. It is noticed that decreased flexible work patterns for the mothers, because many mothers mainly care for children and do household chores.

This study evaluates how the QOL of children and their parents has been affected by changes in the sleep cycles of children with NDDs and stresses caused by mothers’ work patterns during the COVID-19 pandemic. We also explore possible factors that have enabled some of the children and their parents to maintain their QOL.

## Results

### Participant backgrounds

The clinical backgrounds of the children and their parents are shown in Table 1. We recruited 152 parents and their school-aged children with NDDs from the Hachioji area during the COVID-19 emergency. Questionnaires were completed by 136 of 152 parents (89.5%). We surveyed their backgrounds and daily life patterns and assessed the QOL and mental health of the parents and children. The range of participants included 132 mothers (97.0%), two fathers (1.5%), one grandmother (0.7%), and one adult sister (0.7%).

**Table 1:** Clinical background of children and their parents.

Children	Total
Male: female	104:32
Age M ± SD	10.6 ± 2.6
WISC/WAIS FSIQ M ± SD	84.9 ± 15.5
ADHD N, (%)	78 (57.4)
ASD N, (%)	65 (47.8)
SLD N, (%)	9 (6.6)
Alteration of sleep rhythm N, (%)	57 (41.9)
Late bedtime N, (%)	32 (23.5)
Late waking time N, (%)	24 (17.6)
Both late bedtime and waking time N, (%)	1 (0.7)
Use of social media and games; ≥ 5 h N, (%)	27 (19.9)
**Changes in education N, (%)**	
Online school classes available	12 (8.8)
Learning with handouts at home and a few school days	123 (90.4)
Regularly go to school	1 (0.7)
**Parent**	**Total**
Participants; Mother: Father: Other than parents	131: 3: 2
Participants; Age M ± SD	42.4 ± 5.8
Single parent family (only mother family) N, (%)	12 (8.8)
**Mother’s working situation N, (%)**	
Usual working pattern	46 (33.8)
Changed working pattern^†^	59 (43.4)
House keeping	31 (22.8)
Parenting adviser; grandparents N, (%)	61 (44.8)
Parenting adviser; medical, or welfare support organizations available N, (%)	104 (76.5)

(%) Data indicate proportion of each characteristic in every group.

*N* number, *M* mean, *SD* standard deviation, *FSIQ* full scale intellectual quotient, *ADHD* attention-deficit hyperactivity disorder, *ASD* autism spectrum disorder, *SLD* specific learning disorder.

^†^Changed working pattern suggested short working hours, stopped working, tele-work, etc.

There were 79 (58.1%) children with unchanged sleep rhythm, and 57 (41.9%) children with changed sleep rhythm (32 children with late bedtime, 24 children with late bedtime and late waking time, and 1 child with late waking time) compared with the pre-COVID-19 pandemic conditions based on parental observation (Table 1). No children took hypnotics or were newly diagnosed with a sleep disorder during the COVID-19 stay-at-home period. There were also 46 (33.8%) mothers with usual working patterns, 59 (43.4%) mothers with changed working patterns, and 31 (22.8%) mothers who were housewives, compared with those before the COVID-19 pandemic (Table 1). No parents were unemployed during the COVID-19 stay-at-home period.

Table 2 shows the results of the questionnaires. Forty-nine children (36.0%) scored above the CBCL internalizing score cut-off of ≥ 70 points for clinical range, and 38 children (27.9%) scored above the CBCL externalizing score cut-off of ≥ 70 points for clinical range. Sixty-two parents (45.6%) scored above the cut-off of ≥ 16 points (CES-D) for depression; 12 parents (8.8%) were in the top five percent of the general population for high-state anxiety according to the STAI, 13 parents (9.6%) were in the top five percent for high-trait anxiety according to the STAI, 23 parents (16.9%) were in the top five percent for high parenting stress associated with the parental domain of the PSI, and 51 parents (37.5%) were in the top five percent for high parenting stress associated with the children’s domain of the PSI. The median QOL score for children was 72.5 out of 100.0 points on the Kiddo-KINDL^R^ questionnaire, and the median QOL score for parents was 63.8 out of 100 points on the WHOQOL-BREF questionnaire.

**Table 2:** Questionnaires administered children and their parents.

Questionnaire: children	Median, range	N (≥ Cut-off) (%)	Cut-off
Total scores of Kiddo-KINDL	72.5, 41.3–95		–
Physical health	80, 40–100		–
Emotional well-being	80, 40–100		–
Self-esteem	60, 20–100		–
Family	70, 30–95		–
CBCL, internalized index	65.5, 40–93	49 (36.0)	≥ 70
CBCL, externalized index	65.0, 39–91	38 (27.9)	≥ 70
**Questionnaire: parents**			
Total scores of WHO-QOL-BREF	63.8, 29.2–92.3		–
Domain 1 Physical health	63, 6–94		–
Domain 2 Psychological	50, 6–100		–
Domain 3 Social relationships	56, 0–100		–
Domain 4 Environment	56, 19–100		–
CES-D	14.5, 0–56	62 (45.6)	≥ 16
STAI, state	49.0, 22–80	12 (8.8)	Woman: ≥ 64^‡^
			Man: ≥ 66^‡^
STAI, trait	49.0, 23–78	13 (9.6)	Woman: ≥ 65^‡^
			Man: ≥ 66^‡^
PSI, parent domain	115.5, 66–169	23 (16.9)	≥ 137^‡^
PSI, children domain	104.0, 54–152	51 (37.5)	≥ 111^‡^

*N* number, *CBCL* Child behavior checklist, *WISC-IV* Wechsler Intelligence Scale for Children–Fourth Edition, *WHO* world health organization, *QOL* quality of life, *CES-D* center for epidemiologic studies depression scale, *STAI* state-trait anxiety inventory, *STI* parenting stress index.

^‡^Score was above the 95 percentiles.

### QOL changes in children and parents

Supplementary Table S1 compares QOL for families before and during the COVID-19 pandemic. Usual working pattern in mothers and worsening sleep patterns in children were associated with low QOL scores for parents and children. A diminished QOL of the parents was associated with the absence of grandparents, who might provide extra support.

### Factors that protect QOL for parents and children with NDDs

**Figure 1:**
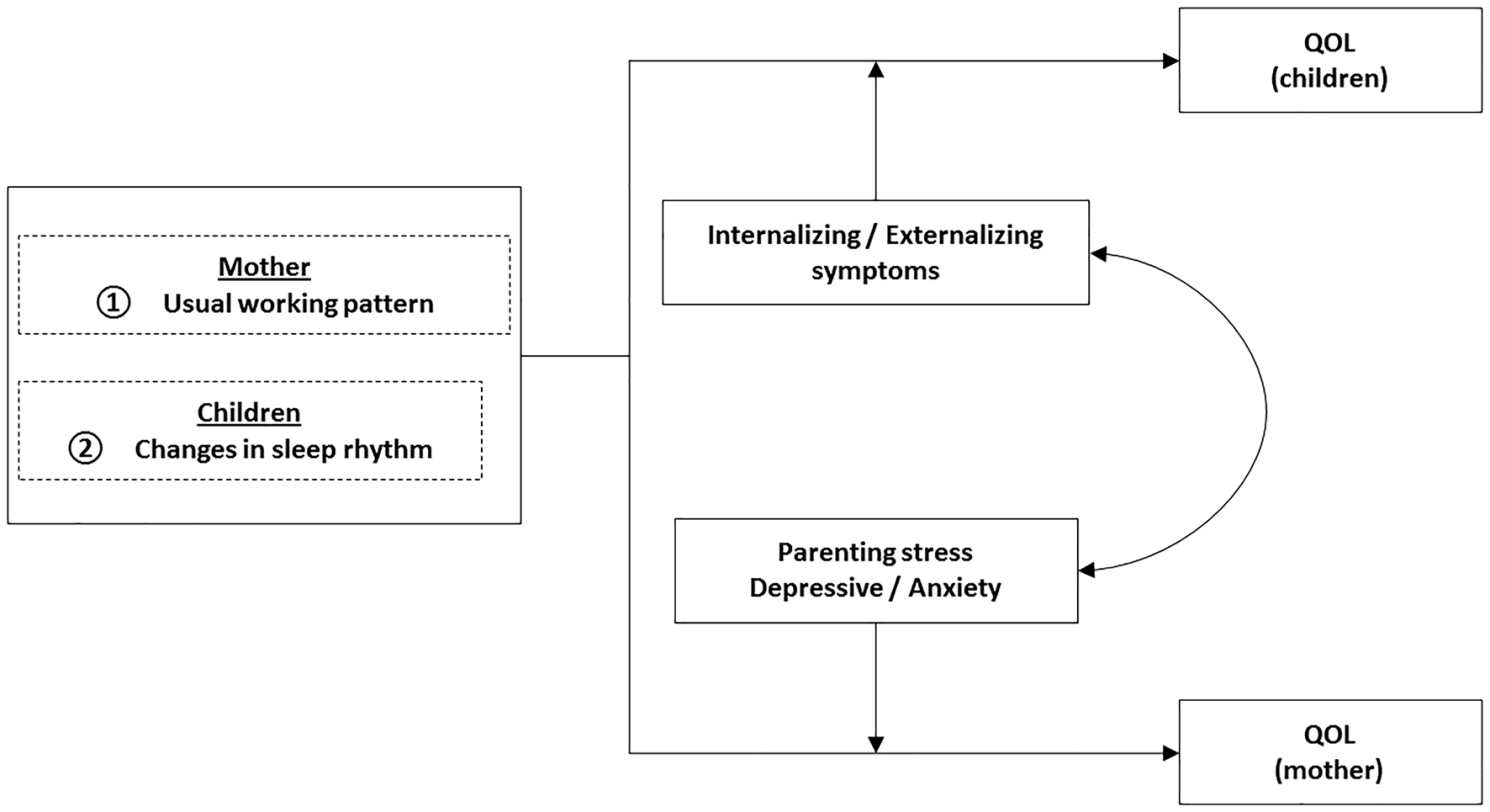
Factors affecting QOL during the COVID-19 stay-at-home period. Children with worsening sleep patterns and mothers with usual working patterns were two factors of a reduced family QOL. In spite of these factors, some families were able to maintain a healthy quality of life with reduced parenting stress, less parental depression and anxiety, and milder internalizing and externalizing symptoms in the children with NDDs. Conversely, increased parenting stress, a severe depressive state and anxiety in parents, and severe internalizing and externalizing symptoms in children deteriorated the QOL of children with NDDs and their parents. Internalizing and externalizing symptoms in children were associated with parental stress, a depressive state, and anxiety in parents.

We investigated the clinical characteristics of children and parents who were able to maintain their QOL even after the profound changes brought about by the COVID-19 pandemic. We analyzed alterations in the children’s sleep rhythms and the mothers’ working patterns as factors for additional analysis (Fig. 1, Supplementary Table S2).

Fifty-seven children suffered from altered sleep patterns; this group was divided into the high-QOL group (> 70: 27 children) and low-QOL group (≤ 70: 30 children); parents were also divided into a high-QOL group (≥ 60: 29 parents) and low-QOL group (< 60: 28 parents). The high-QOL groups, both parents and children, also had lower STAI state- and trait-anxiety scores, parent- and child-domain PSI scores, and internalizing CBCL scores than the low-QOL group. On the other hand, low-QOL children had higher externalizing CBCL scores than high-QOL children, and the low-QOL parents had higher CES-D scores than the high-QOL parents.

The parental QOL was higher when the mother’s job unpredicted flexible work arrangements. For families where there was usual working pattern, children were divided into a high-QOL group (> 70: 21 children) or a low-QOL group (≤ 70: 25 children); and parents were also divided into a high-QOL group (≥ 60: 24 parents) and a low-QOL group (< 60: 22 parents). Both children and parents in high-QOL groups had lower CES-D depressive state scores, STAI state- and trait-anxiety scores, PSI parent domain scores, and CBCL internalizing scores than both in low-QOL group; children in the high-QOL group had lower scores in the PSI children domain in PSI, and externalizing score in CBCL than children in the low-QOL group.

A simple linear regression analysis revealed a relationship between the PSI child-domain and the CBCL total score: *R*^2^ = 0.574, F = 183.295, and *P* < 0.001 (Fig. 1).

## Discussion

We examined the QOL of children with NDDs and their parents during Japan’s COVID-19 stay-at-home period, April 7 to May 25, 2020. The lack of QOL was associated with situations where mothers had little job flexibility, similar to the usual work and commute pattern, and when there was a change in the child’s sleep patterns. The decreased QOL of the parents was also associated with absence of grandparent support. Internalizing and externalizing symptoms in children were strongly associated with parental stress due to the child’s maladaptive behavior.

However, in the children with bad sleep patterns and parents who had similar working patterns, QOL maintenance was associated with less parental stress, less anxiety and depression, and only mild maladaptive behavior in the children. In parents with unusual work and their children, QOL maintenance was also associated with less parental stress, less anxiety and depression, and only mild maladaptive behavior in the children.

### Alterations of child and parent QOL

The COVID-19 pandemic restricted daily life activities and resulted in mandated social distancing measures, which required children to stay at home and to wear masks to prevent infection when going out. Children with NDDs were presumed to be associated more with lifestyle changes and a high level of uncertainty caused by the COVID-19 pandemic than TDC and to have an increased level of maladaptive behaviors^1^. In addition, parents faced additional parenting stress^10^.

A recent online anonymous study on adults showed that the COVID-19 pandemic was negatively associated with mental health (i.e., stress and depression) and QOL^11–13^. A higher risk for increased anxiety and depression was seen in younger women (18–29), unemployed persons, individuals with a prior psychiatric history, and those reporting a greater negative impact on their QOL^11,12^.

As stated above, we found that three factors were associated with decreased QOL of parents and children with NDDs. The first factor was changes in the sleep rhythms of the NDD children. This population was prone to sleep problems, which were associated with QOL, even when there was no outside crisis. Previous studies have shown that sleep problems were associated with the QOL of children with ADHD and ASD^14–16^. Sleep problems in children with NDDs also were associated with parental QOL, stress levels, and physical health^7,17^. A Turkish study during the COVID-19 pandemic indicated that severe sleep orders led to greatly increased ASD symptoms^18^. In our study, changes in the children’s sleep rhythm were associated with decreased QOL of both children with NDDs and their parents.

The second factor that was related to the QOL of parents and children was inflexible mothers’ jobs after the COVID-19 pandemic. Although the mother was still working away from home similar to pre-COVID-19 pandemic, she had an increased burden of household chores and educational demands. It was difficult for the parent to cope with the childcare burden, which had increased due to school closures, compared to a parent who could telecommute, had decreased work hours, or stopped working^8,9^.

The third factor that was related to the QOL of parents was the absence of grandparents, who can play an important role in helping parents with NDD children. One study showed how grandparents who had autistic grandchildren helped the parents understand autistic symptoms^19^. Unfortunately, we cannot comment on whether grandparent support deteriorated or not during the COVID-19 emergency, although we did reveal the presence of a certain proportion of grandparent support during the COVID-19 emergency. On the other hand, according to a survey by the cabinet office in Japan, Japanese grandparents hoped to interact more closely with their grandchildren compared with grandparents in Western countires^20^. It was speculated that Japanese grandparents played more important roles in child care. Another study suggested that one of the factors associated with reduced QOL of parents of children with ASD was the lack of reciprocal social interaction with others, including friends, other family members, or members of the community^6^. Mutual social support might be an important factor for the QOL of parents during the COVID-19 pandemic. Proactively using non-face-to-face support methods, such as online video conferencing platforms, was one way to cope with enforced separation during the COVID-19 pandemic.

In this study, we showed that the reduced QOL of parents and their children during the COVID-19 stay-at-home period was associated with the three aforementioned factors, although it was unclear whether the QOL of parents and children was lower than that before the COVID-19 among all participants.

### Synergistic relationship between maladaptive behavior of children and parenting stress

During the COVID-19 emergency, there was a high proportion of NDD children who were internalizing and externalizing symptoms and also a high proportion of parents suffering from depression and stress (Table 2). The t-scores of CBCL internalizing and externalizing symptoms in children with NDDs were associated with PSI child-domain scores in their parents. The PSI child-domain reflects parental stress associated with the child’s maladaptive behavior; it is possible that increased parental stress was associated with severe internalized and externalized symptoms from the children.

Conversely, lower parental resilience in the face of increased childcare demands was associated with increased internalization and externalization of the symptoms of the children. Previous studies detail similar relationships between the behavioral problems of children with NDDs and parental stress^21,22^. Socially inappropriate and disruptive behaviors evident in children with NDDs are poorly tolerated socially, leading to increased parental stress^21^. Children’s maladaptive behaviors and parenting stress could synergistically be associated with decreased QOL for each group.

### Factors protecting QOL of parents and children with NDDs

The outline of the results was shown in Fig. 1. As mentioned above, two factors were associated with reduced QOL of parents and children: children with poor sleep rhythms and mothers whose jobs were inflexible. However, as stated above, some parents and children maintained QOL during the COVID-19 stay-at-home period.

In previous studies, reduced parental and child QOL has been associated with severe child behavioral difficulties and parenting stress^6,21,23^. Previous studies have reported the experiences of children with NDDs after other natural disasters; the children showed an increased occurrence of mental health problems and reduced adaptive behavior^24,25^. In an Italian study, ASD children had more intense and frequent disruptive behavior during the COVID-19 stay-at-home period^2^. Almost all caregivers with children with ASD confronted increased difficulties in managing daily activities, especially free time^2^. Preliminary data from a study of 3,000 Chinese families during the COVID-19 period showed that parents with ASD children suffered from depression (46.01%), anxiety (44.67%), and stress (44.62%)^4^. Although our study showed that Japanese children with NDDs had high externalizing and internalizing symptoms, the internalizing symptoms had a stronger relationship with QOL in both children and parents. Measures to reduce children's maladaptive behavior and parents’ depression and anxiety were important issues to maintain the QOL of children and parents.

In previous studies, strong social support received after a disaster has been associated with an increased psychological resilience^26^. Access to social supports for children with NDDs and their families during COVID-19 pandemic has also been crucial to address vulnerability factors, guide adjustments in home environments, and apply mitigation strategies to improve coping^3^. There was an urgent need to shift from the conventional care system to the remote delivery of healthcare and NDDs support^3^. To improve the QOL of children with NDDs and their parents, it is necessary to build a dedicated system for education, welfare services, and healthcare with public support.

This study had several limitations. First, the number of patients analyzed in the study was small, and the diagnoses of the NDDs varied. However, all participants were families in Hachioji and its neighboring cities, which are commuter towns. The rate of receiving welfare in Hachioji is similar to the national average, as mentioned in the methods. The families had relatively homogeneous life-behavior patterns. It was estimated that traveling between the city center and the suburbs caused an increased number of COVID-19-infected people in these cities, as in most parts of Japan. Second, it is unclear whether the QOL of parents and children was lower during COVID-19 than before COVID-19, because the QOL of parents and children with NDDs is always significantly lower than the general population^5,6,27^. Third, we cannot comment on whether grandparent support deteriorated or not during the COVID-19 emergency, as mentioned above. Fourth, it was impossible to accurately diagnose sleep disorders because there were no interviews by doctors regarding children’s sleep and no sleep diaries. Fifth, children with moderate or severe intellectual disabilities were excluded in this study. Although we understand the importance of considering QOL of children with intellectual disabilities, we prioritized eliminating the effects of intellectual ability on sleep pattern and mental health in this study. Target age and intellectual level of the children included in this study were decided from the ability of mutual communication in words.

The participants agreed to answer the same questionnaires again after the COVID-19 pandemic ends, and we would like to compare the results between the COVID-19 stay-at-home period and post-COVID-19.

In conclusion, this study discussed the QOL status of children with NDDs and their parents during the COVID-19 stay-at-home period. Decreased QOL of parents and their children was associated with usual working pattern of the mothers and changes in the sleep patterns of the children. Internalizing and externalizing symptoms in children were strongly associated with parental stress due to the child’s maladaptive behavior. QOL maintenance of children and parents was associated with lower parental stress, a milder level of anxiety and depression, and milder maladaptive behavior in the children. There were two factors associated with poor QOL: mothers with job inflexibility and children with changes in sleep patterns.

## Methods

### Data collection

In May 2020, during the COVID-19 pandemic and the subsequent declaration of a state of emergency, we conducted a study of children with NDDs and their parents at the Shimada Ryoiku Center Hachioji: a regional core outpatient clinic where children receive medical examinations, rehabilitation, and psychotherapy. Hachioji is located in the western suburbs of Tokyo. It is a commuter town with a population of 580,000 (population density 3093/km^2^). Low-income households in Hachioji requiring welfare as per the Public Assistance Act accounted for 1.75% of all households, compared with the 1.68% nationwide average^28^.

The age range of children from elementary to senior high school in this study was 6–18 years. The inclusion criteria were (1) children with NDDs (attention-deficit hyperactivity disorder [ADHD], ASD, specific learning disorders [SLD], tic disorders, or other neurodevelopmental disorders), as classified by DSM-5, and (2) school-aged children (from 6 to 18 years old). The exclusion criteria were (1) children with moderate or profound intellectual disabilities (because validation of CBCL in intellectual disability was uncertain in the previous study^29^, we prioritized eliminating the effects of intellectual ability on sleep patterns and mental health in this study) and (2) parents who did not understand the questionnaires written in Japanese.

The government of Japan enforced a state of emergency from April 7 to May 25 and mandated that individuals stay at home, with no activities outside the home. School attendance had been suspended even earlier, beginning March 2 and continuing until May 30. During this period, there were 19 persons infected with COVID-19 in Hachioji City (a 6.8% rate of positivity).

### Assessment tools

One caregiver from each family completed the following questionnaires: (1) an assessment of the parent’s clinical status: the Japanese version of the State-Trait Anxiety Inventory (STAI)^30^, the Center for Epidemiologic Studies Depression Scale (CES-D)^31,32^, the Parenting Stress Index (PSI)^33,34^, and the World Health Organization Quality of Life-BREF (WHOQOL-BREF)^35^; (2) an assessment of the children’s clinical status: the Child Behavior Checklist (CBCL)^36^ and the KINDL^R^ to measure health-related QOL^37,38^; (3) an assessment of daily life during the COVID-19 stay-at-home period based on parent’s perception, including changes in the working hours and commuting habits of the mother or father, availability of parental advisers like grandparents, the children’s education skills, children’s sleep patterns, and amount of time using social media and online games; (4) The parents’ (respondents) and children’s ages and the educational histories of the mother and father. The questionnaires were collected by mail. The children’s NDD diagnoses and intellectual quotient scores were confirmed by the attending physician.

The items of the Kiddo-KINDL^R^ were rated on a five-point Likert scale, and mean scores for each sub-scale and total items were calculated and converted to a 0–100 scale. The averaged values in the Kiddo-KINDL^R^ of four sub-scales (physical well-being, emotional well-being, self-esteem, and family), excluding social contact and school sub-scales, were calculated to evaluate the children’s QOL. The items of the WHOQOL-BREF were also rated on a five-point Likert scale, and the raw total score for each of the four domains (physical health, psychological, social relationships, and environment) was converted to a score (0–100), following the manual^39^. The total items score of the WHOQOL-BREF (0–130) was converted to a 0–100 scale. The averaged values of the 26 items in the WHOQOL-BREF were calculated to evaluate the parent’s QOL. All QOL scores showed better QOL at higher scores.

### Statistical analysis

A one-way analysis of variances (ANOVAs) on the Kiddo-KINDL^R^ and WHOQOL-BREF was performed to examine factors affecting the QOL of children and their parents. We used subject demographic data as grouping variables for the ANOVAs such as the presence or absence of alternative working patterns (changes in working hours and commuting) of the mother and the presence or absence of sleep changes in the children. A one-way ANOVA was also performed on the parental QOL to examine the presence or absence of grandparents who played roles as parental advisers.

The clinical characteristics of children and parents who could maintain the QOL despite having low-QOL factors were examined. The two items associated with poor QOLs of parents and children were selected from the items for which significant differences were obtained by the above ANOVA (*P* < 0.05). Children and parents with specific factors that reduced QOL were divided into groups with high QOL and low QOL, respectively, based on the median; one-way ANOVA was performed to examine group differences (both high- and low-QOL groups) in the t-scores of internalized and externalized indexes on CBCL, child-domain scores and parent-domain scores of PSI, state-anxiety scores and trait-anxiety scores of STAI, and CES-D scores.

In addition, the relationship between the PSI child-domain and CBCL total scores were assessed using simple linear regression analysis.

All statistical analyses for this study were performed using JMP software, Version 9.0.3 Copyright 2010 SAS Institute Inc. SAS and all other SAS Institute Inc. product or service names are registered trademarks or trademarks of SAS Institute Inc., Cary, NC, USA.

### Ethical approval and informed consent

This study followed the guidelines of the Declaration of Helsinki and was approved by the institutional review board of the Shimada Ryoiku Center Hachioji (Shimahachi-2001). For all participants, dedicated staff explained the study while maintaining social distance in a well-ventilated large room, and the parent agreed to his/her participation and provided written informed consent.

## Supplementary Information

Supplementary Information

## Data Availability

Data are available upon reasonable request.
