# Supplementary material for: The quality of life of children with neurodevelopmental disorders and their parents during the Coronavirus disease 19 emergency in Japan
Source: Sci Rep. 2021 Feb 15;11:3042. doi: 10.1038/s41598-021-82743-x (PMC7884401; doi:10.1038/s41598-021-82743-x)
Supplement: Supplementary file 1 — Supplementary Information [file 41598_2021_82743_MOESM1_ESM.docx]

**The quality of life of children with neurodevelopmental disorders and their parents during the Coronavirus disease 19 emergency in Japan**

Riyo Ueda^1,2*^, Takashi Okada^1^, Yosuke Kita^3,4^, Yuri Ozawa^2^, Hisami Inoue^2^, Mutsuki Shioda^2^, Yoshimi Kono^2^, Chika Kono^2^, Yukiko Nakamura^2^, Kaoru Amemiya^2^, Ai Ito^2^, Nobuko Sugiura^2^, Yuichiro Matsuoka^2^, Chinami Kaiga^2^, Masaya Kubota^2^, Hiroshi Ozawa^2^

## ^1^ Department of Developmental Disorders, National Institute of Mental Health, National Center of Neurology and Psychiatry, Tokyo, Japan

^2^ Department of Child Neurology, Shimada Ryoiku Center Hachioji, Tokyo, Japan

^3^ Mori Arinori Center for Higher Education and Global Mobility, Hitotsubashi University, Tokyo, Japan

^4^ Cognitive Brain Research Unit (CBRU), Faculty of Medicine, University of Helsinki, Helsinki, Finland

## *Corresponding Author:

## Riyo Ueda, MD, PhD,

## Department of Developmental Disorders, National Institute of Mental Health, National Center of Neurology and Psychiatry, 4-1-1 Ogawahigashi-Cho, Kodaira, Tokyo 187-8553, Japan

## Tel: +81 42 341 2711; Fax: +81 42 346 2158

E-mail address: [uedariyo@ncnp.go.jp](mailto:uedariyo@ncnp.go.jp)

Supplementary Table S1. Causes of worsening children and parent QOL

| **QOL of Children** | **Presence (**M ± SD**)** | **Absence (**M ± SD**)** | ***P* value** |  |
| --- | --- | --- | --- | --- |
| Mother with usual working pattern | 69.5 ± 7.6 | 73.7 ± 11.6 | *0.028 |  |
| Changes in sleep rhythm of children | 69.0 ± 12.6 | 74.6 ± 8.0 | *0.002 |  |
| **QOL of Parent** | **Presence** (M ± SD) | **Absence** (M ± SD) | **P value** |  |
|  |  |  |  |  |
| Mother with usual working pattern | 57.3 ± 7.7 | 62.2 ± 8.8 | *0.002 |  |
| Changes in sleep rhythm of children | 57.2 ± 8.5 | 62.9 ± 8.2 | *<0.001 |  |
| Parenting adviser; grandparents | 62.7 ± 8.1 | 58.7 ± 8.9 | *0.008 |  |

* P *va*lue <0.05

QOL; quality of life; N, number; M, mean; SD, standard deviation; ADHD, attention deficit hyperactivity disorder; ASD, autism spectrum disorder; SLD, specific learning disorder; FSIQ, full scale intellectual quotient

Supplementary Table S2. Results of related factors for maintaining QOL

1. Alteration of children’s sleep rhythm

| **QOL of Children** | **Preservation** (M ± SD) | **Aggravation** (M ± SD) | ***P* value** |
| --- | --- | --- | --- |
| CES-D | 18.5 ± 12.2 | 20.9 ± 9.6 | 0.422 |
| STAI, state | 49.4 ± 12.7 | 56.4 ± 10.3 | *0.026 |
| STAI, trait | 49.3 ± 12.6 | 55.6 ± 10.9 | *0.049 |
| PSI, parent domain | 108.8 ± 20.9 | 128.4 ± 20.1 | *0.016 |
| PSI, children domain | 99.2 ± 16.5 | 120.0 ± 18.0 | *<0.001 |
| CBCL, internalizing index | 63.5 ± 11.6 | 73.5 ± 7.9 | *<0.001 |
| CBCL, externalizing index | 62.6 ± 9.6 | 71.7 ± 9.4 | *<0.001 |
| **QOL of Parent** | **Preservation** (M ± SD) | **Aggravation** (M ± SD) | ***P* value** |
| CES-D | 15.1 ± 7.3 | 24.9 ± 12.0 | *<0.001 |
| STAI, trait | 49.3 ± 11.0 | 57.3 ± 11.7 | *0.010 |
| STAI, state | 47.2 ± 10.9 | 58.7 ± 10.4 | *<0.001 |
| PSI, parent domain | 106.9 ± 21.0 | 132.7 ± 15.5 | *<0.001 |
| PSI, children domain | 103.5 ± 20.4 | 117.5 ± 17.2 | *0.007 |
| CBCL, internalizing index | 65.6 ± 11.6 | 72.3 ± 9.3 | *0.021 |
| CBCL, externalizing index | 66.2 ± 9.9 | 68.6 ± 11.1 | 0.4 |

1. Mother with usual working pattern

| **QOL of Children** | **Preservation** (M ± SD) | **Aggravation** (M ± SD) | ***P* value** |
| --- | --- | --- | --- |
| CES-D | 13.7 ± 10.8 | 20.0 ± 10.0 | *0.040 |
| STAI, state | 47.7 ± 11.2 | 54.5 ± 9.7 | *0.033 |
| STAI, trait | 57.3 ± 9.5 | 47.3 ± 9.0 | *<0.001 |
| PSI, parent domain | 104.9 ± 19.3 | 132.3 ± 18.6 | *<0.001 |
| PSI, children domain | 98.8 ± 16.3 | 118.6 ± 16.2 | *<0.001 |
| CBCL, internalizing index | 61.0 ± 7.7 | 72.1 ± 5.9 | *<0.001 |
| CBCL, externalizing index | 63.4 ± 5.6 | 70.9 ± 7.7 | *<0.001 |
| **QOL of Parent** | **Preservation** (M ± SD) | **Aggravation** (M ± SD) | ***P* value** |
| CES-D | 11.2 ± 6.5 | 22.5 ± 11.1 | *<0.001 |
| STAI, state | 46.9 ± 7.5 | 55.6 ± 11.9 | *0.005 |
| STAI, trait | 46.2 ± 7.6 | 58.8 ± 9.1 | *<0.001 |
| PSI, parent domain | 109.7 ± 22.2 | 129.0 ± 20.5 | *0.004 |
| PSI, children domain | 104.9 ± 21.8 | 113.8 ± 15.0 | 0.112 |
| CBCL, internalizing index | 64.0 ± 9.3 | 69.8 ± 7.3 | *0.023 |
| CBCL, externalizing index | 66.3 ± 7.3 | 68.6 ± 8.0 | 0.313 |

* P value <0.05

QOL; quality of life; N, number; M, mean; SD, standard deviation; ADHD, attention deficit hyperactivity disorder; ASD, autism spectrum disorder; SLD, specific learning disorder; FSIQ, full scale intellectual quotient; CES-D, Center for Epidemiologic Studies Depression Scale; STAI, state trait anxiety inventory; STI, parenting stress index; CBCL, child behavior checklist
